# On the Use of Chloral Hydrate Enemata

**Published:** 1879-02-20

**Authors:** 


### *ON THE USE OF CHLORAL-HYDRATE ENEMATA.*

Dr. Starcke, of Berlin, has a paper on the employment of chloral-hydrate enemata in the *Berliner Klinische Wochenshcrift* for August last. He observes that there are great prejudices, especially in England, against the continued use of chloral, occasioned probably by the not unfrequent misadventures occurring in connection with its use in habitual drunkards. Last year Dr. Starcke himself fell ill of a chronic gastric catarrh, with great acidity of the contents of the stomach and considerable emaciation and prostration. The principal and most distressing symptom, however, was persistent insomnia, only half an hour to an hour's sleep being obtained at night. At the suggestion of his colleagues Dr. Starcke resorted to the use of chloral, but the irritable state of the stomach forbade its use by the mouth, and hence he determined to take it *per rectum.* An aqueous five per cent. solution of chloral was warmed to about 95° Fahr., of which he injected first ten grammes, and after a quarter of an hour a further quantity of ten grammes, so that in all one gramme (fifteen and a half grains) of chloral were thus taken. This was in a few minutes followed by a feeling of warmth, comfort and repose, and lastly by sound sleep, which lasted uninterruptedly for five hours. In this manner Dr. Starcke continued the injection of chloral for five months, taking in all 120 grammes of the drug. Decided convalescence set in after almost the very first dose, which was followed every morning by a sense of vigor and a desire for food, without any headache or other discomfort. Nor did the efficacy of the dose of chloral diminish, and latterly even half the quantity, *i. e.*, 0.5 gramme, was sufficient. Frequently the attempt was made to obtain sleep without resorting to the chloral, but in vain, until within the last month, when Dr. Starcke found he could discontinue it altogether. This employment of chloral *per rectum* has decided advantages in cases of gastric irritability. Dr. Starcke tried twice to take it by the mouth, and each time it was after a few minutes rejected, and no sleep ensued. The absence of all unpleasant results when administered by the rectum is doubtless due to its undergoing no decomposition, as is generally the case when it comes in contact with the contents of the stomach. Of course the drug should be absolutely pure. The sensation of burning and tenesmus which at first follows an injection, may be materially obviated by well oiling the nozzle of the syringe. And since the site of the tenesmus is chiefly in the region of the sphincter, contact of the chloral solution with this part of the gut should be avoided by passing the injection pipe as high up as possible. And if the injection is made by one's self the position on knees and elbows will be found the most convenient. It is also of consequence that the solution should be complete, and that it should be warmed to the temperature of the body; also that the dose required is a moderate and even small one as compared with that usually given by the mouth. Dr. Starcke has subsequently used chloral in the same way in various cases and with the same uniformly safe and favorable results. It seems especially applicable in the case of aged people, and in no case need the dose exceed one gramme (fifteen and a half grains).—*London Med. Record.*

